# A Low Gastric pH Mouse Model to Evaluate Live Attenuated Bacterial Vaccines

**DOI:** 10.1371/journal.pone.0087411

**Published:** 2014-01-29

**Authors:** Karen E. Brenneman, Crystal Willingham, Jacquelyn A. Kilbourne, Roy Curtiss 3rd, Kenneth L. Roland

**Affiliations:** 1 Center for Infectious Diseases and Vaccinology, The Biodesign Institute, Arizona State University, Tempe, Arizona, United States of America; 2 School of Life Sciences, Arizona State University, Tempe, Arizona, United States of America; Facultad de Medicina, Uruguay

## Abstract

The low pH of the stomach serves as a barrier to ingested microbes and must be overcome or bypassed when delivering live bacteria for vaccine or probiotic applications. Typically, the impact of stomach acidity on bacterial survival is evaluated in vitro, as there are no small animal models to evaluate these effects in vivo. To better understand the effect of this low pH barrier to live attenuated *Salmonella* vaccines, which are often very sensitive to low pH, we investigated the value of the histamine mouse model for this application. A low pH gastric compartment was transiently induced in mice by the injection of histamine. This resulted in a gastric compartment of approximately pH 1.5 that was capable of distinguishing between acid-sensitive and acid-resistant microbes. Survival of enteric microbes during gastric transit in this model directly correlated with their in vitro acid resistance. Because many *Salmonella enterica* serotype Typhi vaccine strains are sensitive to acid, we have been investigating systems to enhance the acid resistance of these bacteria. Using the histamine mouse model, we demonstrate that the in vivo survival of *S.* Typhi vaccine strains increased approximately 10-fold when they carried a sugar-inducible arginine decarboxylase system. We conclude that this model will be a useful for evaluating live bacterial preparations prior to clinical trials.

## Introduction

Live recombinant attenuated *Salmonella* vaccines (RASVs) for humans are typically derived from *Salmonella enterica* serovar Typhi. However, *S.* Typhi is host restricted to humans, thus preclinical vaccine research and development relies on the closely related *Salmonella enterica* serovar Typhimurium as a model, since *S.* Typhimurium causes a disease in mice very similar to human typhoid [1]. Use of the *S*. Typhimurium mouse model has led to a large number of important insights into *Salmonella* pathogenesis and methods to attenuate these bacteria. However, there are a number of important differences between the two serovars and the ways they interact with their respective hosts that, if not understood and addressed, can result in failure at the clinic. One such area that has been relatively overlooked is the impact of the gastric environment on vaccine viability and subsequent interactions with the host.

The gastric acid produced by the stomach serves an important barrier function in preventing host infection by enteric pathogens [2]–[4]. The pH of the gastric environment varies depending on the host species, but is routinely very low [5]. The low pH rapidly inactivates or kills the vast majority of ingested microbes [6]. To cope with the challenge that a low pH gastric environment poses, enteric microbes have evolved a variety of acid resistance strategies. These include such systems as the highly potent urease of *Helicobacter pylori*, amino acid decarboxylases such as glutamate, arginine and lysine decarboxylases and the acid tolerance response found in γ-proteobacteria [7]–[12]. Induction of these systems, usually by exposure to moderately acidic conditions, renders the infecting microbe temporarily resistant to low pH [12]–[14]. The level of protection from low pH provided by these systems depends on the properties of the individual enzymes and the microenvironment of the low pH compartment, but generally, these are highly successful strategies for transiting through the gastric environment.

Problems arise when desirable microbes, such as RASVs, need to be introduced into humans via the oral route. There is a fundamental difference between *S.* Typhi and *S.* Typhimurium in that *S.* Typhi is more acid-sensitive than *S.* Typhimurium [15]. There is also a fundamental difference in the gastric biology of mice and humans. Prior to immunization, mice and humans fast (usually 4–6 hours) to empty the upper portion of the gastrointestinal tract and reduce variations in vaccine invasion into M cells of the gut associated lymphoid tissue (GALT) due to the presence of food. In mice, fasting has the added benefit of increasing gastric pH. The normal murine gastric pH is around 3.0, and rises to 4.0 following a fast [16]. In humans, however, the normal stomach pH drops below 2.0 during fasting conditions [17], [18]. In the context of an oral immunization, the fasted mouse stomach poses a mild challenge, while the fasted human stomach is hostile enough to eradicate most (if not all) of the incoming vaccine cells.” To further complicate matters, RASVs are not usually cultured under conditions that induce acid resistance, thus the cells enter the host unprepared for the challenge the gastric environment presents [19]. RASVs may also contain mutations that render them more sensitive to acidic pH than wild-type strains [15], [20]. These problems make it difficult to reliably predict the survival of RASVs following inoculation and may hamper our understanding of *Salmonella* pathogenesis and RASV immunogenicity. Thus, a mouse model that more closely mimics the low gastric pH of humans may provide additional insights to guide the development and formulation of RASVs for human use.

The problem gastric transit poses for RASVs has by no means been overlooked. Vaccines are usually administered in conjunction with a means to protect the cells from gastric acid, such as an antacid [21]–[24] or in a gel-coated capsule [25], [26]. These formulations are effective, but they prevent the target microbe from experiencing the low pH environment of the stomach and thus the cells do not receive important positional signals that aid them in preparing to colonize the intestine and invade host tissues [27]–[31]. To address this issue, we have developed a regulated acid-resistance strategy using arginine decarboxylase that dramatically increases the survival of acid-sensitive *Salmonella* Typhi vaccines strains at pH 3.0 and 2.5 in vitro [32]. However, for this system to be effective in a vaccine context, it must increase survival not only in vitro, but also during in vivo gastric transit.

In mice, the subcutaneous administration of histamine results in a rapid and significant increase in hydrochloric acid secretion by the parietal cells of the stomach via signaling through the H_2_ receptors [33]–[36]. Although originally designed to mimic acid reflux in humans, this model may also be suitable for microbial infection and immunization scenarios, as it was previously used to establish the importance of low gastric pH as a barrier to infection [4]. Thus, the purpose of this study was two-fold. The first goal was to determine the suitability of the histamine mouse model as a means to mimic the low pH environment of the human stomach and gain insight into the impact of the in vivo gastric environment on the survival of RASVs and other enteric microbes. The second goal was to use the histamine mouse model to evaluate the in vivo survival of attenuated *S.* Typhi strains containing a rhamnose-regulated arginine decarboxylase acid resistance system. This system significantly improves survival during in vitro low pH challenge [32]. Thus, we hypothesized that the presence of this system would also increase the ability of acid-sensitive strains of *S.* Typhi to reach the intestinal tract in vivo. We found that survival of a variety of wild-type bacteria in this low gastric pH mouse model strongly correlated with their in vitro acid resistance profile and that the arginine decarboxylase system provided approximately a 10-fold competitive advantage in the low pH gastric environment for acid-sensitive strains of *S.* Typhi.

## Materials and Methods

### Animal use and ethics statement

This study was approved by the Arizona State University Institutional Animal Care and Use Committee (IACUC). All animals were housed in accordance with American Association for Laboratory Animal Care (AALAC) standards, provided unlimited access to food and water, and handled in accordance with the Animal Welfare Act and Institutional Animal Care and Use Committee (IACUC) regulations. Experiments involving animals were conducted in a facility fully accredited by the Association for Assessment and Accreditation of Laboratory Animal Care International (Unit #000765) and an assurance is on file with the Office for Laboratory Animal Welfare (#A3217-01). Experiments were planned and conducted utilizing the three R's (reduce, replace and refine) which included environmental enrichment, veterinary oversight and the use of appropriate analgesics and anesthesia when appropriate. For surgical procedures, mice were anesthetized using pentobarbital. Euthanasia at the completion of experiments was carried out by carbon dioxide asphyxiation or cervical dislocation while under pentobarbital anesthesia.

### Bacterial strains, plasmids and culture conditions

The bacterial strains and plasmids used in this study are listed in **Table 1**. For routine use, strains were propagated in LB medium (supplemented with 0.1% glucose) with shaking at 200 rpm at 37°C [37]. For acid resistance and gastric transit assays, cells were also grown in tryptic soy broth (TSB) (BD Biosciences, Franklin Lakes, NJ, USA) with 0.4% glucose at 37°C under static anaerobic conditions or in minimal EGA medium (E medium containing 0.4% glucose and 0.1% casamino acids [32]) with shaking at 200 rpm at 37°C. For *S.* Typhi strains containing the rhamnose-dependent arginine decarboxylase system, 0.1% rhamnose was supplied in the growth medium. *S.* Typhi EGA medium cultures were supplemented with 20 µg/ml L-tryptophan, 22 µg/ml L-cysteine, and 0.1% casamino acids. Media for the growth of Δ*aroD* strains χ11548 and χ11568 were additionally supplied with 50 µg/ml L-phenylalanine, 20 µg/ml L-tyrosine, 2 µg/ml *ρ*-aminobenzoic acid and 2.5 µg/ml 2, 3-dihydroxybenzoate. For antibiotic selection, streptomycin and kanamycin were used at 30 µg/ml, while ampicillin was used at 100 µg/ml. All chemicals were purchased from Sigma-Aldrich (St. Louis, MO, USA) or Thermo Fisher Scientific (Pittsburgh, PA, USA) unless otherwise indicated.

**Table 1:** **Bacterial strains and plasmids used in this study.**

Strain	Description/Genotype^a^	Reference or Source
*Escherichia coli* O157:H7 278F2	Wild type	[63]
*Shigella flexneri* 2457T	Wild type	[64]
*Salmonella enterica* serovar Typhimurium UK-1	Wild type	[65]
*Salmonella enterica* serovar Typhimurium LT-2	Wild type, RpoS^-^	[51], [66]
*Vibrio cholerae* C6709	Wild type El Tor, Inaba	[67]
*Salmonella enterica* serovar Typhi Ty2	Wild type, RpoS^-^	[68], [69]
χ11548	*S.* Typhi Ty2 Δ*aroD1299*	[32]
χ11568	*S.* Typhi Ty2 Δ*aroD1299* ΔP_adiA276_::TT *rhaSR* P_rhaBAD_ *adiA* Δ(P_adiY_-*adiY*-P_adiC_)-*119 adiC*	[32]
χ8444	*S.* Typhi Ty2 Δ*phoPQ23*	[32]
χ11622	*S.* Typhi Ty2 Δ*phoPQ23* ΔP_adiA276_::TT *rhaSR* P_rhaBAD_ *adiA* Δ(P_adiY_-*adiY*-P_adiC_)-*119 adiC*	[32]
χ11118	*S.* Typhi Ty2 ΔP_fur81_::TT *araC* P_araBAD_ *fur*	[32]
χ11623	*S.* Typhi Ty2 ΔP_fur81_::TT *araC* P_araBAD_ *fur* ΔP_adiA276_::TT *rhaSR* P_rhaBAD_ *adiA* Δ(P_adiY_-*adiY*-P_adiC_)-*119 adiC*	[32]
**Plasmid**		
pWSK129	pSC101 *ori*, Kan^R^	[70]
pGB2	pSC101 *ori*, Str/Spc^R^	[71]
pGEN-*luxCDABE*	p15A *ori*, Amp^R^, *luxCDABE*	[38]

a In genotype descriptions, the subscripted number refers to a composite deletion and insertion of the indicated gene. P, promoter; TT, T4 ip III transcription terminator; *ori*, origin of replication; Kan^R^, kanamycin resistance; Str/Spc^R^, streptomycin/spectinomycin resistance; Amp^R^, ampicillin resistance.

### Histamine mouse model and measurement of murine intragastric pH

Six week old, female BALB/c mice (Charles River Laboratories, Wilmington, MA, USA) were fasted without food or water for 6 h prior to the start of the experiment. Mice received the histamine H_1_-receptor antagonist chlorpheniramine (0.3 mg/kg) subcutaneously to prevent allergy/anaphylaxis symptoms. Gastric acid secretion was induced by subcutaneous injection of histamine dihydrochloride (10 mg/kg) [33], [34]. For intragastric pH measurements, mice were anaesthetized with pentobarbital (50 mg/kg intraperitoneally), stomachs were excised and the pH of the gastric contents was immediately measured using an Orion 9863BN Micro pH electrode (Thermo Fisher Scientific). Care was taken during pH measurement to fully immerse the pH probe in the gastric contents without contacting the stomach mucosa. Mice were euthanized immediately after pH measurements were taken.

### Visualization of *S.* Typhi in the gastrointestinal tract

As a means to visualize the location of the microbial inoculum following oral immunization, the plasmid pGEN-*luxCDABE* was introduced into *S.* Typhi Ty2. pGEN-*luxCDABE* encodes the *Vibrio* luciferase genes and emits a strong photon signal (5×10^5^ CFU emit a visible signal) [38]. This plasmid was stable for >25 generations in *S.* Typhi. Mice were treated to induce low gastric pH as described above and were orally inoculated with 1×10^9^ CFU of *S.* Typhi Ty2(pGEN-*luxCDABE*) 50 min after histamine injection. Bacterial cells were administered in 20 µl PBS (pH 7.4) supplemented with 0.1% casamino acids. At 30, 60 and 90 min after inoculation, mice were euthanized via cervical dislocation and the GI tract excised and perforated to increase oxygen availability during the imaging process. Luminescent bacteria were detected using the IVIS Lumina pre-clinical in vivo imaging system (Xenogen, Alameda, CA, USA) using luminescence settings with a 300 s exposure.

### In vitro acid resistance assay

Acid resistance was determined essentially as described previously [39], [40] with the following modifications. Wild-type strains were propagated either under normal inoculation conditions (grown to an optical density (OD_600_) of 0.9 in LB broth with 0.1% glucose with aeration) or under acid resistance-inducing conditions (in a static anaerobic TSB culture with 0.4% glucose for 17 h). Cultures were normalized to the same OD_600_ value, then pelleted and washed once in EG medium, pH 7.0 containing no growth supplements [41]. Cells were pelleted a second time and resuspended at a density of 1×10^9^ CFU/ml in EG medium at pH 3.0 containing 0.1% casamino acids. Because the wild-type strains use a variety of decarboxylase enzymes to resist low pH, casamino acids were supplied in order to provide all strains with the same combination of amino acids. Low pH challenge was conducted at 37°C and samples were collected immediately after resuspension (t = 0) and hourly for 4 h. Samples were serially diluted and plated onto LB agar to assess viability during challenge.

### Gastric transit of wild-type enteric strains

Strains were grown under identical conditions as those used in the acid resistance assay with two differences. First, strains used in the gastric transit assays contained the low copy number plasmid pWSK129 to allow for precise quantitation of strain numbers in the non-sterile environment of the gastrointestinal tract. Second, following the completion of growth, strains were pelleted and resuspended in PBS [42] at a density of 5×10^10^ CFU/ml. Murine gastric pH was lowered by histamine injection as described above and groups of 5 mice were orally inoculated with 1×10^9^ CFU of each strain 50 min after the administration of histamine. The average gastric pH at the time of inoculation was 1.46±0.25. Mice were euthanized 1 h after inoculation and the entire small intestine was removed, homogenized and serially diluted. Samples were plated onto LB with kanamycin to determine the number of viable bacteria present following low pH gastric transit.

### Competition assay for acid-resistant and acid-sensitive *S.* Typhi strains

The *S.* Typhi competition assay was performed the same as the gastric transit assays with the following exceptions. First, competitor strains contained one of two low copy number plasmids – either pWSK129 or pGB2. No difference in gastric survival was observed between strains containing these plasmids (data not shown); however, to rule out any effect of antibiotic resistance on gastric transit, each gastric passage experiment was repeated with the plasmids switched. Second, following resuspension at 5×10^10^ CFU/ml, competitor strains were mixed to create an inoculation material with equivalent numbers of each strain. The cells of the inoculum were suspended in PBS containing 1 mM L-arginine. After gastric transit, samples of homogenized intestinal material were plated simultaneously onto LB agar with either streptomycin or kanamycin to enumerate the number of each strain present. Data are expressed as the competitive index for the pair of strains. The number of acid-resistant microbes present was divided by the number of acid-sensitive microbes and then normalized by the initial inoculation ratio for each group.

### Statistical analyses

Statistical calculations were performed using GraphPad Prism version 6.00 for Windows (GraphPad Software, La Jolla, CA, USA). Data are presented as the geometric mean ±95% confidence interval, with the exception of pH measurements which are presented as the mean ± SEM. 2-way ANOVA was used to compare the performance of the fasted and histamine mouse models over the entire strain set, and to compare the difference between acid-adapted and unadapted cells. To determine if the % survival of different strains in a particular model was different, the non-parametric Kruskal-Wallis test was used. For the correlation between survival in the mouse models and survival during in vitro challenge, the log_10_ of the geometric mean number of CFU that survived 1 h in vivo was plotted against the log_10_ of the geometric mean number of CFU that survived 2 h at pH 3.0. Linear regression analysis was used to calculate the best fit line and r^2^ value. Competitive indices were analyzed by the Wilcoxon signed rank test to determine whether the median value differed significantly from 1.000.

## Results

### Optimization of the histamine mouse model for bacterial gastric transit

**Figure 1:**
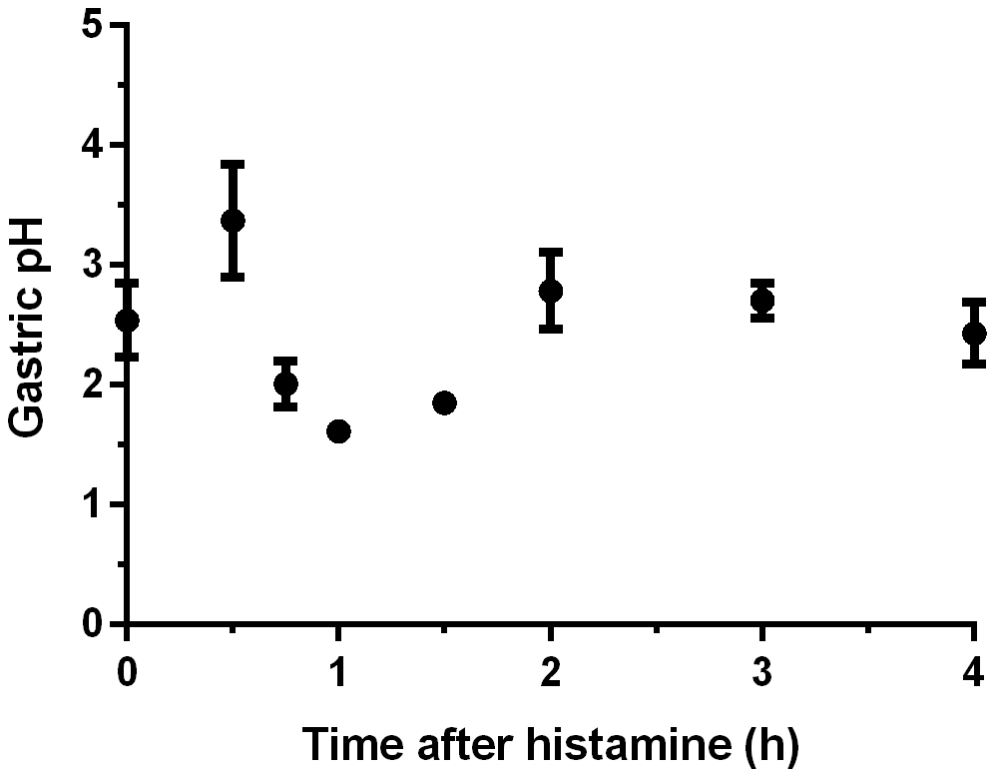
Gastric pH following histamine injection. Following a 6 h fast, mice were injected at time 0 with 10/kg histamine. The pH of the gastric contents was monitored for 4 h post histamine injection. Data shown are the mean and standard error of the mean of at least five mice per time point.

Previous work with the histamine mouse model has shown that maximal secretion of gastric acid occurs 45 min after the administration of histamine [34]. However, this was reported as the amount in µEq of HCl secreted from the mucosal surface and not as the total gastric pH. To evaluate and validate the histamine mouse model as a model for microbial gastric transit, it was necessary to know the length of time required for the entire contents of the stomach to acidify and the duration of the gastric pH minimum so that an appropriate window for oral inoculation could be selected. Thus, our first experiment monitored the gastric pH for 4 h after induction of gastric acid secretion via histamine injection (**Figure 1**). The mean initial gastric pH of mice that had been fasting for 6 h at time 0 was 2.54±0.31. The gastric pH began to decrease 45 min after histamine injection (mean pH = 2.01±0.19), but did not reach a minimum until 60 min (mean pH = 1.61±0.03). The mean pH remained low at 90 min (1.84±0.08), but had returned to the initial value by 2 h (2.78±0.32). Based on these results, we chose to administer oral inoculations to mice 50 min after the injection of histamine to ensure that the incoming bacteria would enter a fully acidified gastric compartment.

**Figure 2:**
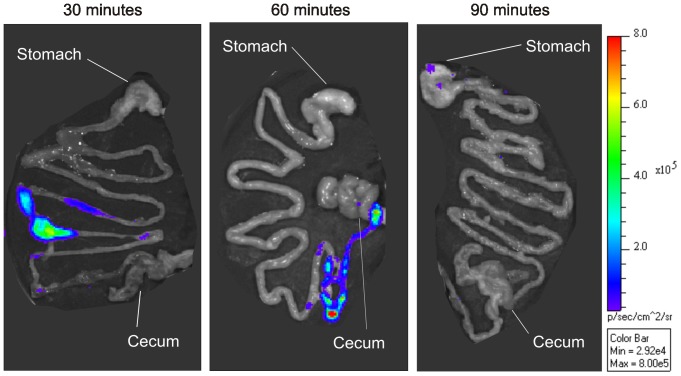
Visualization of the bacterial inoculum in the gastrointestinal tract via light emission. Low gastric pH was induced in mice by histamine injection prior to inoculation with 1×10^9^ CFU of *S.* Typhi Ty2(pGEN-*luxCDABE*). At 30, 60 and 90 min post-inoculation, the gastrointestinal tract was removed and examined for the production of light by luciferase. A visible signal is equivalent to approximately 5×10^5^ CFU. Luminescence is reported as the number of photons detected per s per square cm. Images shown are representative results from groups of seven mice.

The other experimental parameter investigated was the length of time needed for the orally administered bacteria to reach the terminal ileum, which is the primary site of invasion for *Salmonella*. In order to visually identify the location of the inoculated bacteria, the pGEN-*luxCDABE* plasmid was introduced into *S.* Typhi Ty2. Any site containing greater than 10^5^ viable CFU will emit a visible signal, so although the location of every microbe administered could not be determined, the luciferase system encoded by the plasmid was sufficient to detect the bolus of the inoculum. Within 30 min of oral inoculation, the majority of bacteria had already transited the stomach and reached the upper portion of the small intestine (**Figure 2**). By 60 min, the bacteria had reached the terminal ileum, immediately prior to the cecum. After 90 min, the bacteria had presumably entered the cecum, but were no longer detectable, due either to their dilution into the cecal contents or a lack of bioluminescence resulting from a prohibitively low oxygen tension in the cecum. A faint visible signal was observed in the stomachs of some mice. Plating indicated that although a small portion of the inoculated microbes remained in the stomach (approximately 5x10^3^ CFU), the vast majority of bacteria exited to the gastrointestinal tract (data not shown). Based on these results, we selected 1 h after inoculation as the most opportune time to recover the test bacteria from the gastrointestinal tract.

### Enteric bacteria unprepared for low pH challenge survive gastric passage in fasted but not histamine mice

If the histamine mouse model creates a low pH gastric barrier similar to the one found in humans, then survival during transit through the gastric compartment should correlate with the ability of a microbe to resist low pH. In other words, the more acid-resistant a particular pathogen is, the greater the number of bacteria that survive low pH gastric transit. To test this, we selected a panel of wild-type enteric bacteria with differing levels of pH tolerance (**Table 1**). *Escherichia coli* O157:H7 and *Shigella flexneri* were selected as highly acid-resistant strains. The *E. coli* strain is capable of sustained survival at pH 2.0, while *Shigella* is resistant to pH 2.5 [39], [43], [44]. Virulent *Salmonella enterica* serovar Typhimurium strains such as UK-1 do not survive below pH 3.0 unless cultured under conditions that induce acid resistance [43], [45]. We also included *S.* Typhimurium LT-2, which has a defect in the acid tolerance response and exhibits reduced survival at pH 3.0 [27]. Acid-sensitive strains, *Vibrio cholerae* and *Salmonella enterica* serovar Typhi, were also included. *V. cholerae* is highly sensitive to low pH challenge [46]. While not as sensitive as *V. cholerae*, *S.* Typhi strains generally have a much lower capacity to resist low pH challenge than *S.* Typhimurium [15], [47].

**Figure 3:**
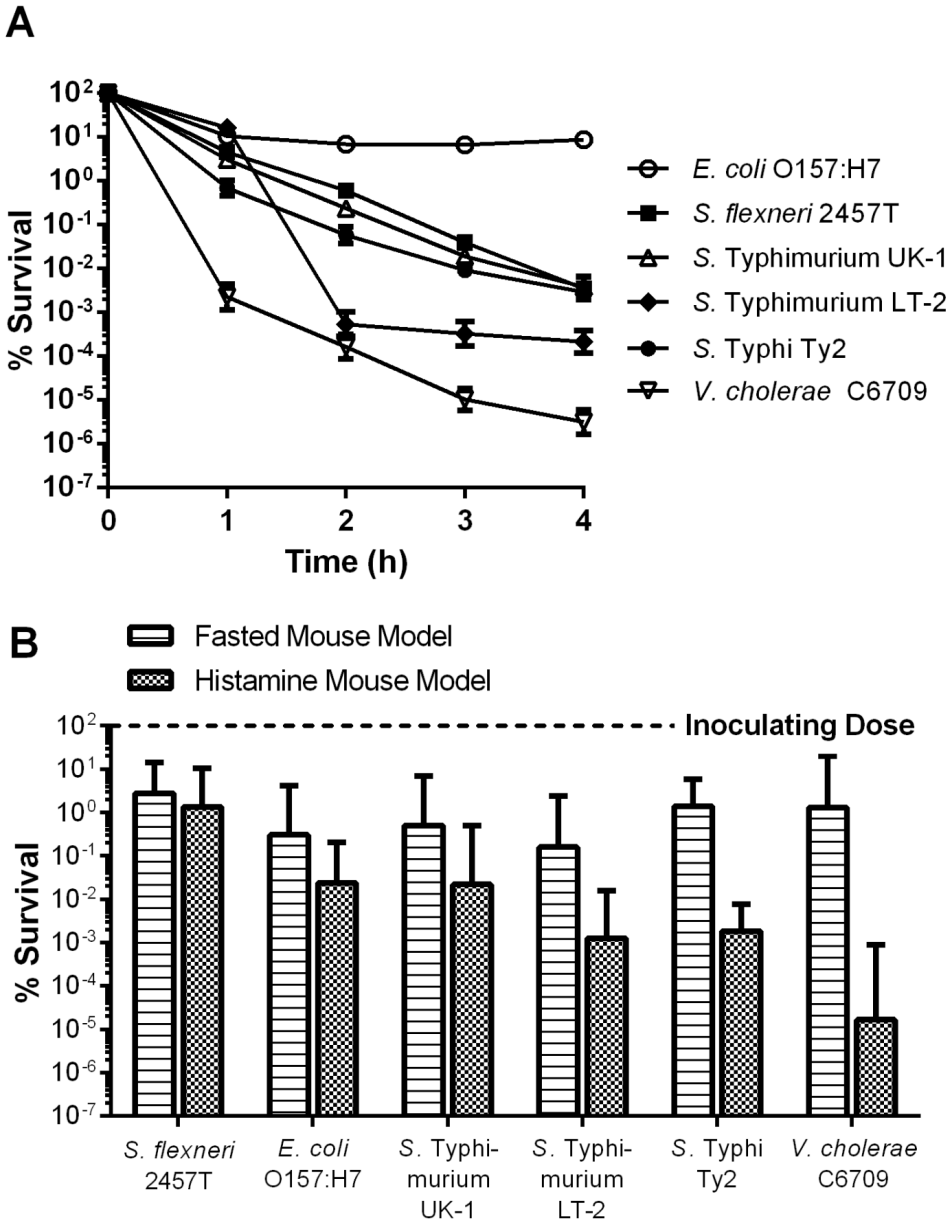
Survival of strains cultured under non-acid resistance inducing conditions. Wild-type enteric strains were grown in LB medium to late-log phase under aerobic conditions. **(A)** Cells were challenged in EG medium (pH 3.0) containing 0.1% casamino acids. Survival during EG medium challenge was assayed hourly for 4 h by plating onto LB agar. Data shown are the mean and SEM of three independent experiments. **(B)** Mice were either fasted for 6 h (fasted mouse model) or fasted and low gastric pH was induced by histamine injection (histamine mouse model) and then inoculated with 10^9^ CFU of each strain. Sixty min after inoculation, mice were euthanized and the entire small intestine removed and homogenized. Strain survival was assayed by plating onto LB agar containing kanamycin. Data are expressed as the percent of initial inoculum recovered (% survival). The geometric mean and 95% confidence interval of two independent experiments (8 mice total) is depicted.

Cells were cultured under routine conditions in LB broth (unprepared for low pH challenge), and their survival during in vitro pH 3.0 challenge was compared to their survival during gastric transit through fasted or histamine-treated mice (**Figure 3**). During in vitro challenge, *E. coli* O157:H7 exhibited essentially no decrease in viability over 4 h, while the viability of *S. flexneri* and the *S.* Typhimurium strains declined gradually during the experiment (**Figure 3A**). *S.* Typhi survived well for the first hour, but a rapid loss in viability was observed after that. The acid-sensitive *V. cholerae* strain exhibited the lowest level of survival. These results are consistent with previously published studies [15], [27], [39], [43]–[46]. When the strains were passed through the gastric compartments of fasted or histamine-treated mice, the models produced significantly different results (*p* = 0.0114) (**Figure 3B**). In fasted mice, no differences in survival were observed between strains (*p* = 0.2757). In contrast, bacterial survival during gastric transit in histamine-treated mice was significantly different between strains (*p*<0.0001). The acid-resistant *S. flexneri* strain had the highest rate of recovery (1.32±5.93%), followed by *E. coli* O157:H7 (2.38±9.24×10^−2^%), while the acid-sensitive *V. cholerae* had the lowest rate of recovery (1.67±21.2×10^−5^%). The acid-sensitive *S.* Typhimurium LT-2 and *S.* Typhi strains also exhibited low rates of recovery in the histamine mouse model (1.27±2.21×10^−3^ and 1.84±3.19×10^−3^, respectively). With the exception of *S. flexneri*, the survival of each strain was significantly lower in the histamine mouse than in the fasted mouse.

### Induction of acid resistance increases survival of enteric bacteria during gastric transit in histamine mice

**Figure 4:**
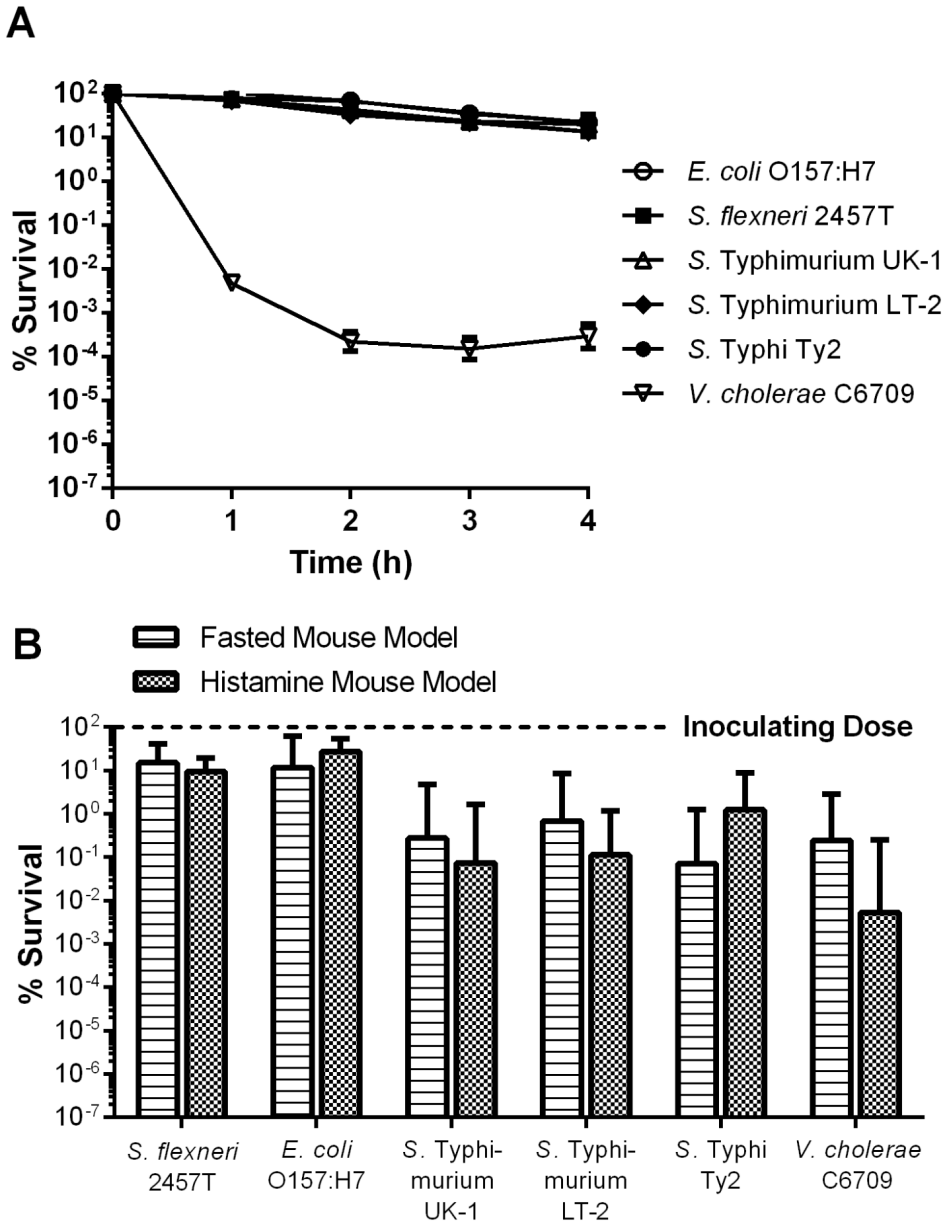
Survival of strains cultured under acid resistance-inducing conditions. Wild-type enteric strains were grown to stationary phase in TSB medium containing 0.4% glucose under anaerobic conditions. **(A)** Cells were challenged in EG medium (pH 3.0) containing 0.1% casamino acids. Survival was assayed hourly for 4 h by plating onto LB agar. Data shown are the mean and SEM of three independent experiments. **(B)** Mice were either fasted for 6 h (fasted mouse model) or fasted and low gastric pH was induced by histamine injection (histamine mouse model) and then inoculated with 10^9^ CFU of each strain. Sixty min after inoculation, mice were euthanized and the entire small intestine removed and homogenized. Strain survival was assayed by plating onto LB agar containing kanamycin. Data are expressed as the percent of initial inoculum recovered (% survival). The geometric mean and 95% confidence interval of two independent experiments (8 mice total) is depicted.

If the differences in survival observed for different strains are indeed due to differences in the ability of the strains to resist low pH, then the induction of acid resistance prior to gastric transit should increase the survival of previously acid-sensitive strains. Thus, the experiment was repeated using cells that had been cultured under anaerobic, low pH conditions to induce acid tolerance and acid resistance (**Figure 4**). Under these conditions, greater than 10% of the initial population of the *E. coli*, *Shigella* and *Salmonella* strains were viable after 4 h of in vitro challenge (**Figure 4A**). This was a significant increase over the survival of unadapted cells (*p* = 0.0011 for LT2, *p*<0.0001 for the other strains). Although growth under acid resistance-inducing conditions improved the ability of *V. cholerae* to survive at the later time points of the challenge, the difference between acid-unadapted and acid-adapted cells at pH 3.0 was not significant (*p* = 0.2363).

**Figure 5:**
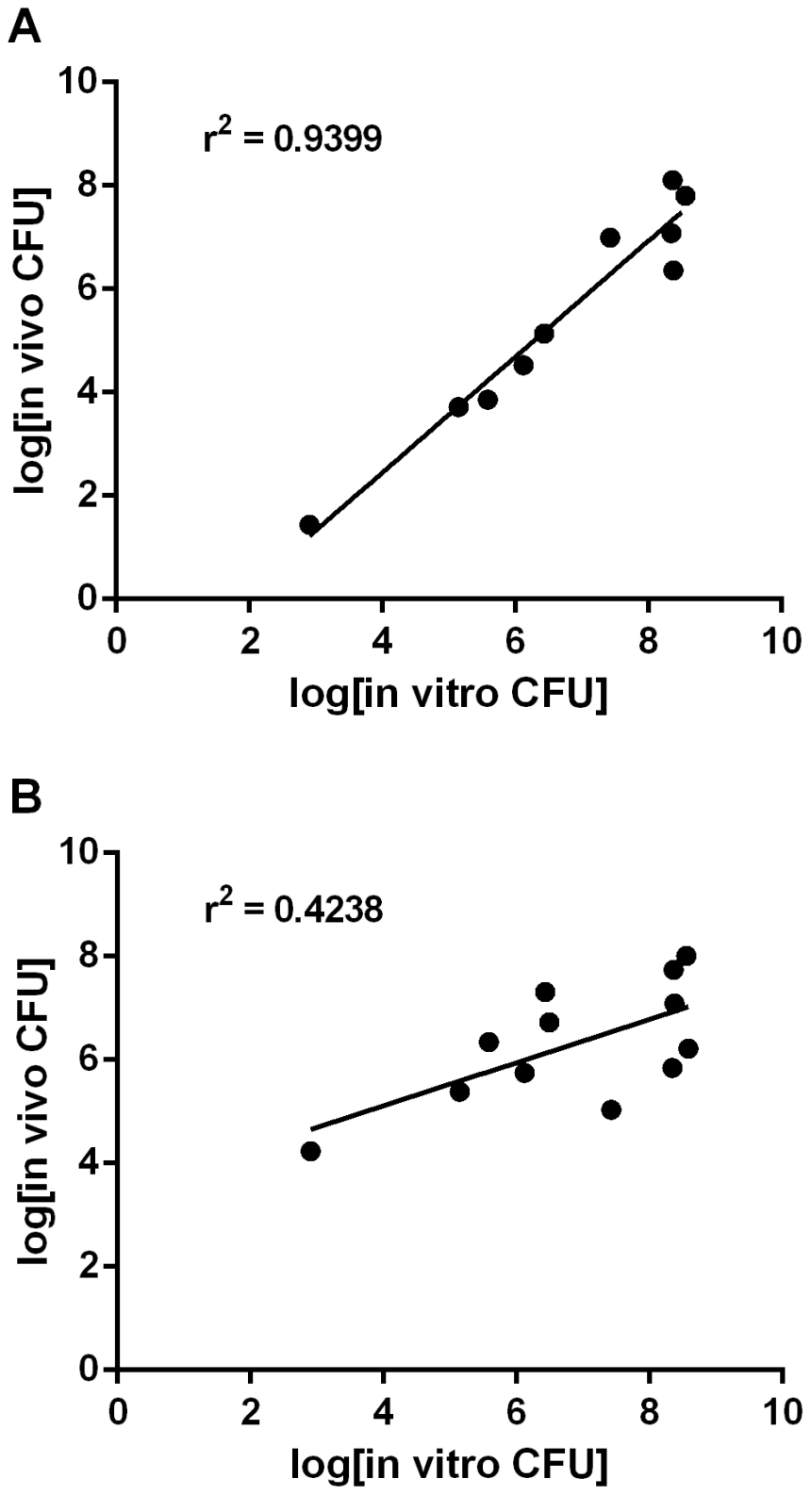
Correlation between in vitro and in vivo survival. The log_10_ of the geometric mean number of CFU that survived in vitro challenge at pH 3.0 for 2 h was plotted against the log_10_ of the geometric mean number of CFU recovered from the intestinal tissue in the **(A)** histamine mouse model or **(B)** fasted mouse model. Linear regression was performed on each data set and the r^2^ value of the best-fit line is depicted for each model.

When acid-adapted strains were administered to mice, survival did not differ between the fasted and histamine-treated mouse models (*p* = 0.4648) (**Figure 4B**). This was a significant improvement in survival during gastric transit over acid-unadapted cells (*p*<0.0001). The greatest increases in survival in vivo were observed with the *E. coli* O157:H7 strain, which increased from 2.38±9.24x10^−2^% to 27.3±15.7% (*p* = 0.0002) and the *S.* Typhi, which increased from 1.84±3.19x10^−3^% to 1.24±4.26% (*p* = 0.0002). Similar to the results observed during in vitro challenge, acid adaptation of *V. cholerae* had no effect on in vivo survival in histamine-treated mice (*p* = 0.0626). To formally assess the ability of the histamine mouse model to discriminate between acid-sensitive and acid-resistant strains, we determined the correlation between survival in vitro during pH 3.0 challenge and survival in vivo (**Figure 5**). The histamine mouse model demonstrated a clear correlation between acid resistance in vitro and the ability to survive gastric transit through an acidified compartment (r^2^ = 0.9399), indicating the power of this model to distinguish between acid-sensitive and acid-resistant microbes (**Figure 5A**). In contrast, the fasted mouse model showed only a weak correlation between acid resistance and survival during in vivo gastric transit (r^2^ = 0.4238) (**Figure 5B**).

### A rhamnose-regulated arginine decarboxylase system increases the survival of acid-sensitive *S.* Typhi strains during gastric transit in histamine mice

**Figure 6:**
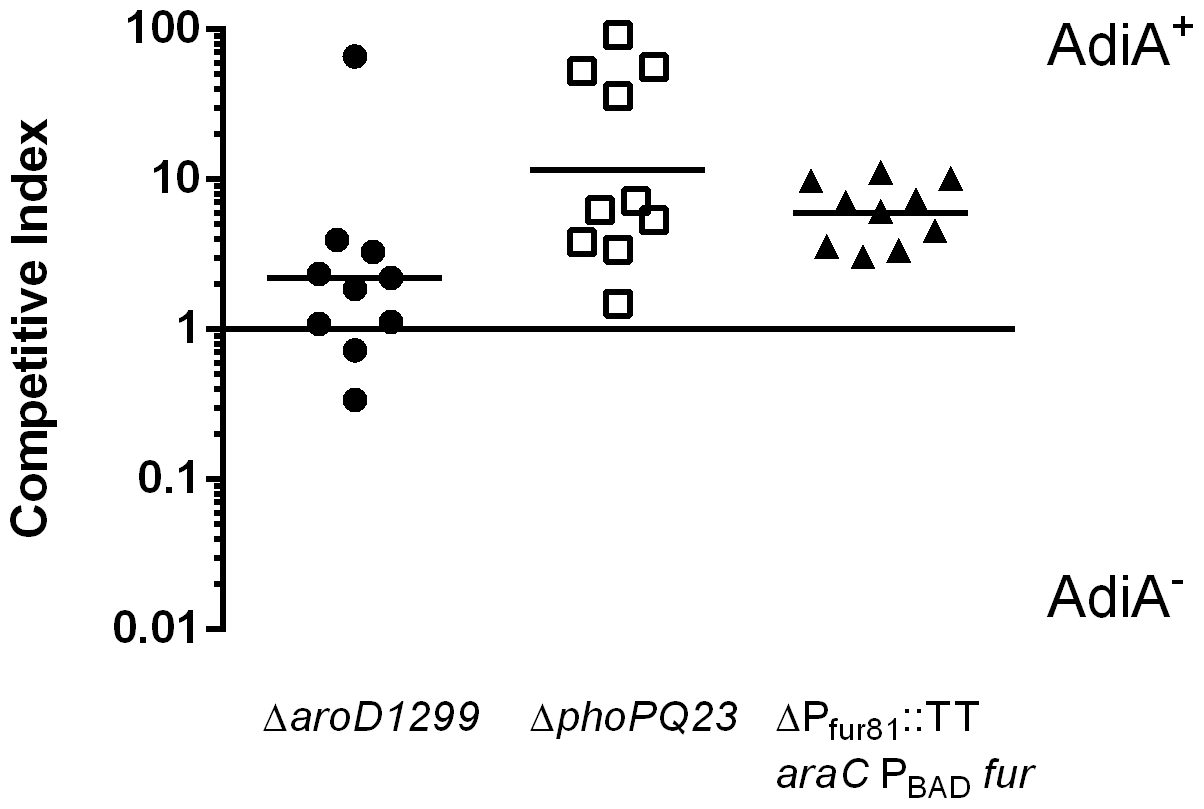
Effect of arginine decarboxylase on the gastric survival of *S.* Typhi. Pairs of attenuated *S.* Typhi strains differing only in their arginine decarboxylase locus were grown to stationary phase in EGA medium under aerobic conditions. Cells were combined in a 1:1 ratio in PBS containing 1 mM arginine. Low gastric pH was induced by histamine injection in mice fasted for 6 h. Mice were inoculated with 10^9^ CFU of each strain. Sixty min after inoculation, mice were euthanized and the entire small intestine removed and homogenized. Strain survival was assayed by plating onto LB agar containing kanamycin or streptomycin. Data shown are the competitive index of the two strains in each mouse with the geometric mean of two independent experiments (10 mice total) indicated as a solid line.

We previously constructed a rhamnose-regulated arginine decarboxylase system in *S.* Typhi, in which the *adiA* and *adiC* genes are placed under the control of the rhamnose-dependent promoter P_rhaBAD_ [32]. Inclusion of rhamnose in the culture medium results in expression of the arginine decarboxylase system, significantly increasing survival during in vitro low pH challenge. This system was introduced into three model *S.* Typhi vaccine strains – Δ*aroD1299* (χ11548), Δ*phoPQ* (χ8444) and ΔP_fur81_::TT *araC* P_BAD_ *fur* (χ11118) (**Table 1**). The mutations in the *phoPQ* and *fur* loci render their respective vaccine strains acid-sensitive, while the mutation in *aroD* has no effect on acid sensitivity. Because the histamine mouse model successfully discriminated between acid-sensitive and acid-resistant bacteria, we used it to evaluate the effectiveness of the rhamnose-regulated arginine decarboxylase system in vivo. We found that the presence of our system provided a competitive advantage during gastric transit (**Figure 6**). The Δ*aroD1299* vaccine strain benefitted the least from the acid resistance system with only a 2.2-fold increase in the survival of χ11568 (AdiA^+^) over the original Δ*aro* parent strain (χ11548). This increase was not significant (*p* = 0.2850). However, survival of the acid-sensitive vaccine strains χ8444 and χ11118 (Δ*phoPQ* and ΔP_fur81_::TT *araC* P_BAD_ *fur*, respectively) was increased by the presence of the rhamnose-inducible arginine decarboxylase system in vivo. In the ΔP_fur81_::TT *araC* P_BAD_ *fur* background, the presence of arginine decarboxylase provided a 6.0-fold increase in survival during gastric transit (*p* = 0.0002). A greater degree of individual variation was observed in the Δ*phoPQ* background, but the inclusion of the regulated arginine decarboxylase system increased survival 11.6-fold in vivo (*p* = 0.0302).

## Discussion

The fasted mouse model and the related bicarbonate-treated fasted mouse model have been used extensively to evaluate the immunogenicity of potential live attenuated *S.* Typhimurium vaccine strains [1], [48]–[50]. However, these models do not take into account differences in the acid sensitivity of potential vaccine strains. Although we observed a lower gastric pH in fasted mice than previously reported (most likely due to differences in the fasting conditions or length), acid-sensitive, acid-adapted and naturally acid-resistant microbes all survived equally well during their transit through the fasted gastric compartment. Although the fasted mouse model is sufficient for the identification and initial characterization of candidate RASVs, results obtained from this model may be misleading because they do not take into account the differences in murine and human gastric biology.

One of the goals of this study was to evaluate the ability of histamine-treated mice to serve as a model system for assessing gastric survival of microbes upon oral inoculation to humans. We found the histamine mouse model to be a reliable means to induce low gastric pH (approximately pH 1.5) for a considerable length of time (45 min). Although the time that the orally administered bacteria spend in the acidified gastric compartment is relatively short (bacteria reach the jejunum within 30 min and are detected in the terminal ileum approximately 1 h after inoculation), we found that even this short exposure to low pH was sufficient to kill a large number of the incoming microbes. Viability of the acid-resistant *E. coli* O157:H7 and *S. flexneri* strains was reduced by 70–90% during gastric transit, while the acid-sensitive *V. cholerae* strain experienced a 100,000-fold decrease in viability.

The low pH gastric barrier created by the histamine mouse model provides an in vivo system that accurately discriminates between acid-sensitive and acid-resistant microbes. Survival during gastric transit in this model directly correlated with the ability of the test microbe to resist low pH. This holds even in the case of the two *S.* Typhimurium strains tested, LT-2 and χ3761 (**Figure 3**). This is of interest, since the two strains have a well-characterized difference in acid resistance, due to their carriage of different rpoS alleles [51]. Cells that were sensitive to acid or unprepared for low pH challenge did not survive as well as cells that had induced acid resistance systems prior to passage. Although survival during gastric transit correlated well with the acid resistance results obtained in vitro, the histamine mouse model routinely returned lower rates of survival than the in vitro assay. This could be due to the difference in pH between the in vitro (3.0) and in vivo (1.5) challenges. However, it is also possible that gastric transit poses additional challenges beyond low pH that are not easily replicated in vitro. In vivo, ingested bacteria must contend with a combination of organic and inorganic acids in the stomach and upper intestinal tract [52] as well as the bile, antimicrobial peptides and other intestinal defense mechanisms [53]–[55]. Thus, when making predictions about the ability of a given microbe to survive oral inoculation, it is important to evaluate in vitro data cautiously, as a moderately acid-sensitive strain in vitro may or may not efficiently transit the stomach in vivo.

Survival of acid-sensitive *S.* Typhi vaccines during gastric transit was improved by the presence of a sugar-inducible acid resistance system (rhamnose-regulated arginine decarboxylase). For both the Δ*phoPQ23* and ΔP_fur81_::TT *araC* P_araBAD_ *fur* strains, the inclusion of this system greatly increased the number of viable bacteria that reached their target tissue for invasion (the terminal ileum). However, the inclusion of the arginine decarboxylase system did not benefit the Δ*aroD1299* mutant to the same degree as the acid-sensitive strains. The reasons for this are not clear, since this strain showed a dramatic increase in acid resistance during in vitro challenge (10,000-fold greater viability after one hour) [32]. One possibility is that growth in minimal medium prior to inoculation may have rendered this nutritional mutant less fit for the challenge of in vivo host interactions. Alternatively, the arginine decarboxylase system may be able to compensate for defects in acid tolerance or acid resistance but be unable to increase acid resistance beyond the wild-type level. There is some evidence for this, as the total number of CFU recovered for both the parent Δ*aroD1299* and acid-resistant Δ*aroD1299* strains was higher than the number recovered from the acid-resistant Δ*phoPQ23* and ΔP_fur81_::TT *araC* P_araBAD_ *fur* strains (data not shown).

In conclusion, the histamine mouse model is a method capable of discriminating in vivo between acid-sensitive and acid-resistant microbes. Survival during gastric transit in this model is directly related to the ability of the ingested microbe to resist low pH - the more acid-resistant a particular microbe is, the greater the number of bacteria that survive gastric transit. This ability makes the histamine mouse model an excellent choice to evaluate not just the delivery of live recombinant attenuated *S.* Typhi vaccines, but any beneficial orally administered microbe or gastric formulation strategy. Many probiotic bacteria have low levels of acid tolerance and require formulations that protect them from exposure to low pH [56]–[59]. This model will allow those formulations to be tested in vivo for efficacy. The histamine mouse model will also permit researchers to explore the natural diversity of acid resistance or sensitivity within a given species or between clinical isolates to determine the effect of acid resistance on strain infectivity and pathogenicity. Many pathogenic enterohemorrhagic *E. coli* strains contain mutations in the *rpoS* and *gadE* genes (GadE is a regulator of the glutamate decarboxylase system) and vary greatly in their ability to resist low pH [8], [60]–[62]. For microbes that exhibit acid sensitivity, the inclusion of the rhamnose-regulated arginine decarboxylase acid resistance system improves the ability of these microbes to reach the intestinal tract. Future studies will focus on determining whether this increase translates into a dose reduction for the vaccine or improved vaccine efficacy.
